# High-Density Geometric Morphometric Analysis of Intraspecific Cranial Integration in the Barred Grass Snake (*Natrix helvetica*) and Green Anole (*Anolis carolinensis*)

**DOI:** 10.1093/iob/obad022

**Published:** 2023-06-05

**Authors:** S Tharakan, N Shepherd, D J Gower, E L Stanley, R N Felice, A Goswami, A Watanabe

**Affiliations:** Department of Anatomy, New York Institute of Technology, College of Osteopathic Medicine, 100 Northern Boulevard, Old Westbury, NY 11568, USA; Department of Genetics, Evolution, and Environment, University College London, Gower Street, London, WC1E 6BT, UK; Life Sciences Division, Natural History Museum, Cromwell Road, London, SW7 5BD, UK; Digital Imaging Division, Florida Museum of Natural History, University of Florida, Gainesville, FL 32611-0001, USA; Department of Genetics, Evolution, and Environment, University College London, Gower Street, London, WC1E 6BT, UK; Life Sciences Division, Natural History Museum, Cromwell Road, London, SW7 5BD, UK; Centre for Integrative Anatomy, Department of Cell and Developmental Biology, University College London, Gower Street, London, WC1E 6BT, UK; Department of Genetics, Evolution, and Environment, University College London, Gower Street, London, WC1E 6BT, UK; Life Sciences Division, Natural History Museum, Cromwell Road, London, SW7 5BD, UK; Department of Anatomy, New York Institute of Technology, College of Osteopathic Medicine, 100 Northern Boulevard, Old Westbury, NY 11568, USA; Life Sciences Division, Natural History Museum, Cromwell Road, London, SW7 5BD, UK; Division of Paleontology, American Museum of Natural History, 79th Street at Central Park West, New York, NY 10024, USA

## Abstract

How do phenotypic associations intrinsic to an organism, such as developmental and mechanical processes, direct morphological evolution? Comparisons of intraspecific and clade-wide patterns of phenotypic covariation could inform how population-level trends ultimately dictate macroevolutionary changes. However, most studies have focused on analyzing integration and modularity either at macroevolutionary or intraspecific levels, without a shared analytical framework unifying these temporal scales. In this study, we investigate the intraspecific patterns of cranial integration in two squamate species: *Natrix helvetica* and *Anolis carolinensis*. We analyze their cranial integration patterns using the same high-density three-dimensional geometric morphometric approach used in a prior squamate-wide evolutionary study. Our results indicate that *Natrix* and *Anolis* exhibit shared intraspecific cranial integration patterns, with some differences, including a more integrated rostrum in the latter. Notably, these differences in intraspecific patterns correspond to their respective interspecific patterns in snakes and lizards, with few exceptions. These results suggest that interspecific patterns of cranial integration reflect intraspecific patterns. Hence, our study suggests that the phenotypic associations that direct morphological variation within species extend across micro- and macroevolutionary levels, bridging these two scales.

## Introduction

Physical and molecular interactions among structures dictate how anatomical structures develop and evolve. The notion of modularity and integration has provided an analytical framework for investigating how these interactions contribute to phenotypic changes during ontogeny and evolution (Olson & Miller 1958; Wagner 1996; Klingenberg 2008, 2014; Goswami et al. 2014; Evans et al. 2022). For example, a more modular structure has been historically thought to promote the evolution of novel and disparate morphologies, where its components are able to evolve semi-autonomously from one another (Wagner & Altenberg 1996). More recent simulation studies have demonstrated that integration could indeed promote the evolution of extreme phenotypes if selection acts along the preferred axes of variation (Goswami et al. 2014; Felice et al. 2018). Given these conceptual scenarios, understanding how patterns of modularity and integration within species translate into clade-wide patterns would help bridge micro- and macroevolutionary trends.

With broad interspecific sampling, recent macroevolutionary studies have examined the links between phenotypic integration and morphological diversification (e.g., Porto et al. 2009; Goswami et al. 2014; Felice & Goswami 2018; Bardua et al. 2020; Fabre et al. 2020; Felice et al. 2020). In concept, these large-scale evolutionary patterns of integration are expected to reflect the genetic control (e.g., pleiotropy), developmental mechanisms, and functional coordination that occur within individuals. Because integration patterns may be altered throughout development (Hallgrímsson et al. 2009), the link between developmental patterns of integration and macroevolutionary patterns could be difficult to evaluate. In comparison, the pattern of modularity and integration between populations of a species sampled at equivalent growth stages (static modularity; Mitteroecker 2009; Klingenberg 2014; Evans et al. 2022), particularly somatically mature individuals, is likely to be more stable in signal due to completion of major anatomical changes, and thus more readily comparable to macroevolutionary trends.

However, most studies on modularity and integration to date have analyzed either evolutionary or intraspecific patterns, without a unified framework to bridge these two levels. Using the same morphometric scheme across intraspecific and interspecific levels, some studies have found generally congruent patterns between select species and clade-wide patterns in caecilians (Marshall et al. 2019) and birds (Mitchell et al. 2021). Within squamates, Urošević and colleagues (2019) used a common landmarking scheme to investigate patterns of modularity across static, ontogenetic, and evolutionary levels within lacertids and found overlapping patterns. In this study, we explore the variational integration within the squamate species *Natrix helvetica* (barred grass snakes) and *Anolis carolinensis* (green anoles). These two taxa were selected because they are collectively abundant in natural history collections and the genera are commonly used as genetic and phylogeographic models (Tollis et al. 2012; Fritz & Schmidtler 2020). Intraspecific patterns of cranial integration have been explored in these two taxa previously (Sanger et al. 2012; Andjelković et al. 2017), but without sampling taxa beyond the genus level to analytically bridge micro- and macroevolutionary scales. Here, the landmark scheme employed on *N. helvetica* and *A. carolinensis* is equivalent to the high-density geometric morphometric data collected for a squamate-wide study (Watanabe et al. 2019). This experimental design allows for proper comparisons between intraspecific and interspecific patterns of integration, while sampling the genera *Natrix* and *Anolis* permit comparisons with results of previous investigations on these two taxa (Sanger et al. 2012; Andjelković et al. 2017). Through these comparisons, we assess (i) differences in intraspecific cranial integration patterns in *N. helvetica* and *A. carolinensis*; and (ii) correspondence of intraspecific integration patterns to respective interspecific patterns in snakes and non-snake extant squamates (hereby “lizards”).

## Materials and methods

### Specimens and imaging

We sampled 29 *N. helvetica* specimens and 41 *A. carolinensis* specimens for this study (Table 1), where one specimen from each taxon was used as template. Specimens exhibiting somatic maturity were selected, with visually intact head. Because the skull of snakes is highly kinetic, specimens that showed symmetric and closed or nearly closed gape were selected to reduce variation in cranial shape due to jaw position. The *Anolis* specimens were scanned using a GE Phoenix v|tome|xm CT Scanner at the University of Florida. *N. helvetica* specimens were imaged with Nikon Metrology HMX ST 225 µCT scanner at the Natural History Museum, London (NHMUK). The segmentation of the cranial elements for both species were performed using Avizo (Thermo Fisher Scientific) to digitally segment and reconstruct the skulls. Upon imaging, many *N. helvetica* specimens were found to unilaterally lack the maxillary bone, presumably removed for another study. However, the remaining side was intact. The resulting PLY mesh files were then imported into GeoMagic Wrap (formerly 3D Systems, now Artec 3D) to remove extraneous objects (e.g., vertebrae, mandible), and we virtually filled in foramina to prevent error in projection of surface semi-landmarks. If the left maxilla was missing from the specimen, then the skull models were mirrored (mirrored specimens specified in Table 1). For *N. helvetica*, we virtually removed teeth from the left side of the pterygoid and palatine bones to eliminate obstructions for the placement of surface semi-landmarks on these elements. Once the teeth were removed, the resulting cavities on the bones were filled using the *Fill Single* tool and its *Tangent* option in GeoMagic Wrap. The *QuickSmooth* function was used to apply global smoothing to the mesh surfaces. The skull models were then exported as PLY mesh files.

**Table 1.** List of specimens sampled for this study. Mirrored specimens refer to those that were digitally mirrored prior to landmarking due to damaged or missing elements on the right side of the skull. Please refer to Watanabe et al. (2019) for information of specimens sampled for the interspecific datasets.

	*Natrix helvetica*		
Specimen no. (NHMUK)	Mirrored?	Sex	Locality
1917.6.22.1 (template)	Yes	M	Droitwich Spa, Worcestershire, UK
96.7.22.3	No	F	New Forest, Hampshire, UK
1961.1648	Yes	F	Portsmouth, Hampshire, UK
1962.306	Yes	F	Europe; United Kingdom; England; Essex
1962.307	Yes	M	Claypitshills Wood, Billericay, Essex, UK
1963.891	Yes	M	Near Ockley, Surrey, UK
1963.980	Yes	M	England, UK [no additional locality data]
1963.981	Yes	F	Devon, UK
1968.1372	Yes	F	Ealing Common, London, UK
1970.312	No	F	Wicken Fen Nature Reserve, Norfolk, UK
1971.1729	No	F	South of Trouillas, Perpignan, France
1971.766	No	M	Hazel Copse, Brasted Chart, Westerham, Kent, UK
1972.1837	No	M	Near Amboise and Bléré, Touraine, France
1973.765	No	F	Near Braunton, Devon, UK
1973.766	No	M	Chivenor, North Devon, UK
1974.4122	No	M	Childerditch Common, Warley, Brentwood, Essex, UK
1974.4123	No	M	Childerditch Common, Warley, Brentwood, Essex, UK
1980.1053	No	M	Round Wood, Kent, UK
1992.543	No	F	Slinfold, Horsham, West Sussex, UK
1896.7.22.1	No	F	New Forest, Hampshire, UK
1908.7.28.1	Yes	F	Jersey, UK
1908.7.28.2	Yes	F	Jersey, UK
1908.7.28.3	Yes	F	Jersey, UK
1908.7.28.4	Yes	M	Jersey, UK
1908.7.28.5	Yes	M	Jersey, UK
1935.5.7.1	Yes	M	Cowes, Isle of Wight, UK
1935.5.7.2	Yes	M	Cowes, Isle of Wight, UK
1947.1.2.99	Yes	M	Barton on Sea, Hampshire, UK
1949.1.2.96	Yes	M	Hamstead Norreys, Thatcham, Berkshire, UK
	*Anolis carolinensis*		
Specimen No.	Mirrored?	Sex	Locality
FMNH 242298 (template)	No	NA	Ouachita Parish, Louisiana, USA
FLMNH 66	Yes	–	Wacahoota Road, Alachua County, Florida, USA
FLMNH 1177–2	No	–	High Springs, Alachua County, Florida, USA
FLMNH 1964–2	No	–	Gainesville, Alachua County, Florida, USA
FLMNH 1964–1	No	–	Gainesville, Alachua County, Florida, USA
FLMNH 2179–1	No	–	Gainesville, Alachua County, Florida, USA
FLMNH 2183–1	No	–	Gainesville, Alachua County, Florida, USA
FLMNH 2505–1	No	–	Gainesville, Alachua County, Florida, USA
FLMNH 2606	No	–	Gainesville, Alachua County, Florida, USA
FLMNH 2900–19	No	–	Gainesville, Alachua County, Florida, USA
FLMNH 2900–18	No	–	Gainesville, Alachua County, Florida, USA
FLMNH 2900–16	No	–	Gainesville, Alachua County, Florida, USA
FLMNH 2900–15	No	–	Gainesville, Alachua County, Florida, USA
FLMNH 2900–13	No	–	Gainesville, Alachua County, Florida, USA
FLMNH 2900–10	No	–	Gainesville, Alachua County, Florida, USA
FLMNH 2900–7	No	–	Gainesville, Alachua County, Florida, USA
FLMNH 2900–6	No	–	Gainesville, Alachua County, Florida, USA
FLMNH 2900–4	No	–	Gainesville, Alachua County, Florida, USA
FLMNH 2900–3	No	–	Gainesville, Alachua County, Florida, USA
FLMNH 2900–1	No	–	Gainesville, Alachua County, Florida, USA
FLMNH 2990–8	No	–	Winter Haven, Polk County, Florida, USA
FLMNH 8025	No	–	State Road 24, Alachua County, Florida, USA
FLMNH 8920–3	No	–	Gainesville, Alachua County, Florida, USA
FLMNH 9080–1	No	–	Gainesville, Alachua County, Florida, USA
FLMNH 9204	No	–	Gainesville, Alachua County, Florida, USA
FLMNH 9560–3	No	–	Paynes Prairie, Alachua County, Florida, USA
FLMNH 9560–4	No	–	Paynes Prairie, Alachua County, Florida, USA
FLMNH 9560–1	No	–	Paynes Prairie, Alachua County, Florida, USA
FLMNH 9561	No	–	State Road 24, Alachua County, Florida, USA
FLMNH 9562	No	–	County Road 346, Alachua County, Florida, USA
FLMNH 9560–2	No	–	Paynes Prairie, Alachua County, Florida, USA
FLMNH 14579	No	–	Gainesville, Alachua County, Florida, USA
FLMNH 14580	No	–	Paynes Prairie, Alachua County, Florida, USA
FLMNH 14678	No	–	Gainesville, Alachua County, Florida, USA
FLMNH 101839	No	–	State Road 26, Alachua County, Florida, USA
FLMNH 101840	No	–	Gainesville, Alachua County, Florida, USA
FLMNH 101841	No	–	State Road 24, Alachua County, Florida, USA
FLMNH 101843	No	–	State Road 121, Alachua County, Florida, USA
FLMNH 101844	No	–	State Road 26, Alachua County, Florida, USA
FLMNH 101848	No	–	Beacon Hill, Bay County, Florida, USA
FLMNH 123200	No	–	Gainesville, Alachua County, Florida, USA

**Abbreviations: FLMNH**, Florida Natural History Museum at University of Florida Herpetology Collections; **FMNH**, Field Museum of Natural History Herpetology Collections; **NHMUK**, Natural History Museum, London, Herpetology Collections.

### Coordinate data

The IDAV Landmark Editor (Wiley et al. 2005) was used to place fixed (discrete) and curved (semi-)landmarks to delineate the following osteological elements: premaxilla, nasal, maxilla, jugal in *A. carolinensis*, frontal, parietal, squamosal in *A. carolinensis*, supratemporal in *N. helvetica*, jaw joint of the quadrate, supra-otoccipital, basioccipital, pterygoid, palatine, and occipital condyle. The jugal is excluded in *N. helvetica* and the snake-wide dataset due to the absence of this bone in snakes. After placing fixed landmarks on both the left and right sides of the skull and curved semi-landmarks on only the right side, the coordinate data were exported as a .pts file. Using R language (R Core Development Team 2022) in RStudio (RStudio Team 2020), we read the .pts files and subsampled the curve semi-landmarks (Botton-Divet et al. 2016; Bardua et al. 2019). The fixed and curve (semi-)landmark scheme is identical to the one used previously for a squamate-wide analysis of cranial integration (Watanabe et al. 2019). The surface (patch) semi-landmarks were placed onto the mesh surface using the *placePatch* function in the *Morpho* R package (Schlager 2017) using *N. helvetica* (NHMUK 1917.6.22.1) and *A. carolinensis* (FMNH 242298) specimens as templates. With the exception of surface semi-landmarks on the pterygoid and palatine bones for *N. helvetica*, these templates contain the same number of surface semi-landmarks as the hemispherical template used previously for the squamate-wide data (Watanabe et al. 2019). These template specimens were not included in the subsequent analysis to maintain equivalency in landmarking procedure across analyzed specimens. In the previous study, surface semi-landmarks were not placed on the pterygoid and palatine bones in snakes due to the presence of teeth, which would lead to inconsistent placement of points. As mentioned above, we virtually removed the palatal teeth and closed the alveoli in GeoMagic Wrap to enable the placement of surface semi-landmarks on the pterygoid and palatine bones in *N. helvetica*.

### Shape data

**Fig. 1.**
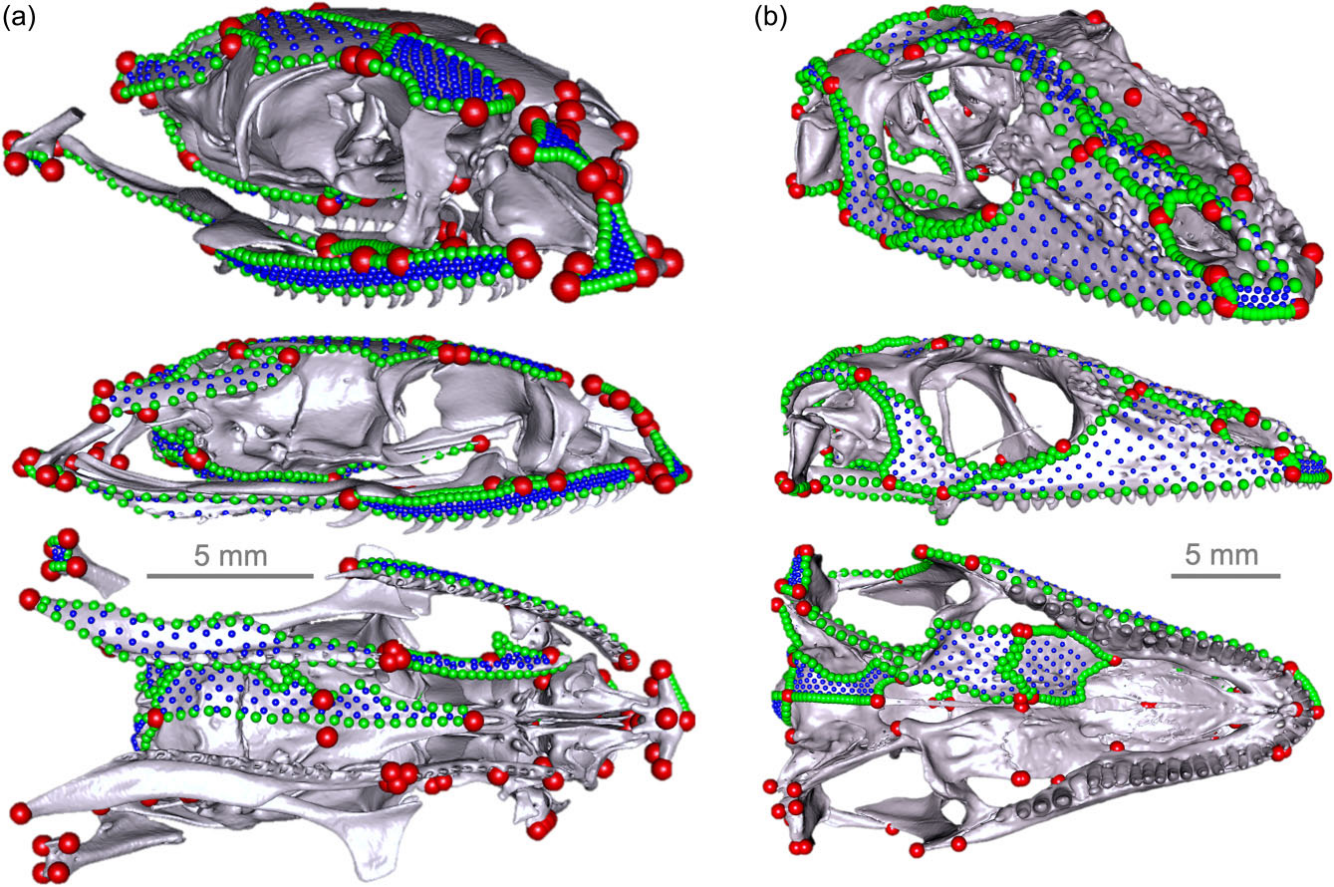
Landmarking scheme used in this study on skull reconstructions of templates (a) *Natrix helvetica* (NHMUK 1917.6.22.1) and (b) *Anolis carolinensis* (FMNH 242298) in oblique (top), lateral (middle), and ventral (bottom) views. Red, green, and blue points are fixed, curve, and surface (patch) (semi-)landmarks, respectively. The dorsal part of the quadrate bone in *N. helvetica* specimens were digitally excised during patching to ensure accurate mapping of surface semi-landmarks on the supratemporal bone. Teeth on palatine and pterygoid teeth in *N. helvetica* were also digitally removed to allow for characterization of the osteological surface of these bones.

Prior to aligning the coordinate data, we used the *mirrorfill* function in the *paleomorph* R package to mirror the curve and surface semi-landmarks placed only on the right side of the skull based on position of bilateral and midline fixed landmarks. This allows creation of artificially bilateral coordinate data to minimize artifacts of aligning one-sided data for bilaterally symmetric structures (Cardini 2016, 2017; Bardua et al. 2019). Following the mirroring process, we performed generalized Procrustes analysis with sliding semi-landmarks, while minimizing total bending energy (Gunz et al. 2005; Gunz & Mitteroecker 2013) on the bilateral coordinate data. Once the generalized Procrustes analysis was performed, semi-landmarks and fixed landmarks on the left-side were removed. Following the curve subsampling and patching process, intraspecific coordinate data of 28 *N. helvetica* specimens (i.e., with the template specimen removed) included 41 fixed, 535 curve, and 549 surface (semi-)landmarks, while those of 40 *A. carolinensis* comprised 47 fixed, 595 curve, and 580 surface (semi-)landmarks (Fig. 1; Table 2). Prior to main analysis, we used the *procD.lm* function in the *geomorph* R package (Adams & Otárola-Castillo 2013) to check for potential biases in our dataset due to surgical removal of one of the maxillae in some of the *Natrix* specimens. The results indicated a lack of significant skull shape differences between *Natrix* with and without intact maxillae (*R*^2^ = 0.0196; *P* = 0.808).

**Table 2.** Landmark (LM), curve semi-landmarks (curve sLMs), and surface semi-landmark (surface sLMs) scheme used for shape analysis. The landmarks are based on Watanabe et al. (2019) for “lizards” and snakes, with the inclusion of surface sLMs for the pterygoid for palatine bones for snakes. LMs and sLM on the jugal were removed for the *Natrix helvetica* dataset because of the lack of this bone in snakes.

Region	No. surface sLMs	LM	Landmark definition	No. curve sLMs
Premaxilla	39	1	Right anterior-most median point of premaxilla	1→2: 10
		2	Right posterior-most median point of premaxilla	2→3: 10
		3	Right dorsal point on ventral premaxilla-maxilla suture	3→4: 5
		4	Right ventral point on ventral premaxilla-maxilla suture	4→1: 10
Nasal	42	5	Right anteromedial-most point of nasal	5→6: 10
		6	Right posteromedial most point of nasal	6→7: 10
		7	Right posterolateral most point of nasal	7→8: 10
		8	Right anterolateral point that meets the naris	8→5: 10
Maxilla	92	9	Right premaxilla-maxilla-naris junction on maxilla	9→10: 10
		10	Right posteromedial point of the maxilla	10→11: 20
		11	Right posterior-most point of the maxilla	11→12: 20
		12	Right anteroventral point of maxilla-nasal suture	12→13: 5
		13	Right anterodorsal point of maxilla-nasal suture	13→9: 10
Jugal	31	14	Right anterior jugal-orbital margin junction	14→15: 20
		15	Right posterodorsal point of the jugal	15→16: 20
		16	Right posterior-most point of jugal	16→14: 20
Frontal	86	17	Right anteromedial point of frontal	17→18: 10
		18	Right posterior medial-most point of frontal	18→19: 10
		19	Right posterolateral point of frontal meeting parietal	19→20: 10
		20	Right anterolateral corner of frontal	20→17: 10
Parietal	34	21	Right anterior median point of parietal	21→22: 20
		22	Right posterior median point	22→23: 10
		23	Right posterolateral point	23→24: 20
		24	Right anterolateral point meeting frontal	24→21: 10
Squamosal/supratemporal	19	25	Right anterior-most point	25→26: 10
		26	Right posterodorsal (medial) most point	26→27: 10
		27	Right posteroventral (lateral) most point	27→25: 10
Jaw Joint	18	28	Right anteromedial point of mandibular articular process	28→29: 5
		29	Right posterolateral point of mandibular articular process	29→30: 5
		30	Right posteromedial point of mandibular articular surface	30→31: 5
		31	Right anterolateral point of mandibular articular surface	31→28: 5
Supra-	67	32	Right dorsal median point of supraoccipital	32→33: 10
Otooccipital		33	Right dorsal median point of foramen magnum	33→34: 10
		34	Right ventral median point of foramen magnum (basion)	34→35: 10
		35	Right ventrolateral most point of oto-basioccipital suture	35→36: 10
		36	Right ventrolateral point of paroccipital process	36→32: 20
Basioccipital	58	37	Right median basioccipital-occipital condyle junction	37→38: 20
+		38	Right anterior-most median point of (basi)sphenoid	38→39: 10
Basisphenoid		39	Right medial sphenoid-pterygoid junction or process	39→40: 20
		40	Right postero-lateral most point of oto-basioccipital suture	40→37: 10
Pterygoid	39	41	Right medial anteroventral point of pterygoid	41→42: 10
		42	Right posterior-most point of pterygoid	42→43: 20
		43	Right lateral anteroventral point of pterygoid	43→41: 20
Palatine	33	44	Right posteroventro-medial point of palate	44→45: 10
		45	Right posteroventro-lateral point of palate	45→46: 20
		46	Right lateral ventral palate-maxilla junction	46→47: 10
		47	Right medial ventral palate-maxilla junction	47→44: 20
Occipital condyle	22	34	Right ventral median point of foramen magnum (basion)	34→37: 5
		37	Right median basioccipital-occipital condyle junction	37→34: 10

### Analyses

Analyses were performed using R v4.1.0 (R Core Development Team 2022). To visualize the pattern of cranial shape variation, we created morphospaces based on the first two principal components (PCs). The strength of integration within and between cranial elements was calculated through the covariation-ratio (CR) method (Adams 2016) and EMMLi, a maximum-likelihood approach (Goswami & Finarelli 2016). Greater ρ (rho) value from EMMLi analysis and CR value indicate greater correlation and covariation. We assessed the relationship between cranial shape to log-transformed centroid size using *procD.lm* function in *geomorph* R package on shape against log-transformed centroid size. For a quantitative comparison of intra- and interspecific integration patterns, a correlation analysis was conducted on CR and EMMLi values between *N. helvetica* and snake-wide data, and between *A. carolinensis* and lizard-wide data.

## Results

### Morphospace

**Fig. 2.**
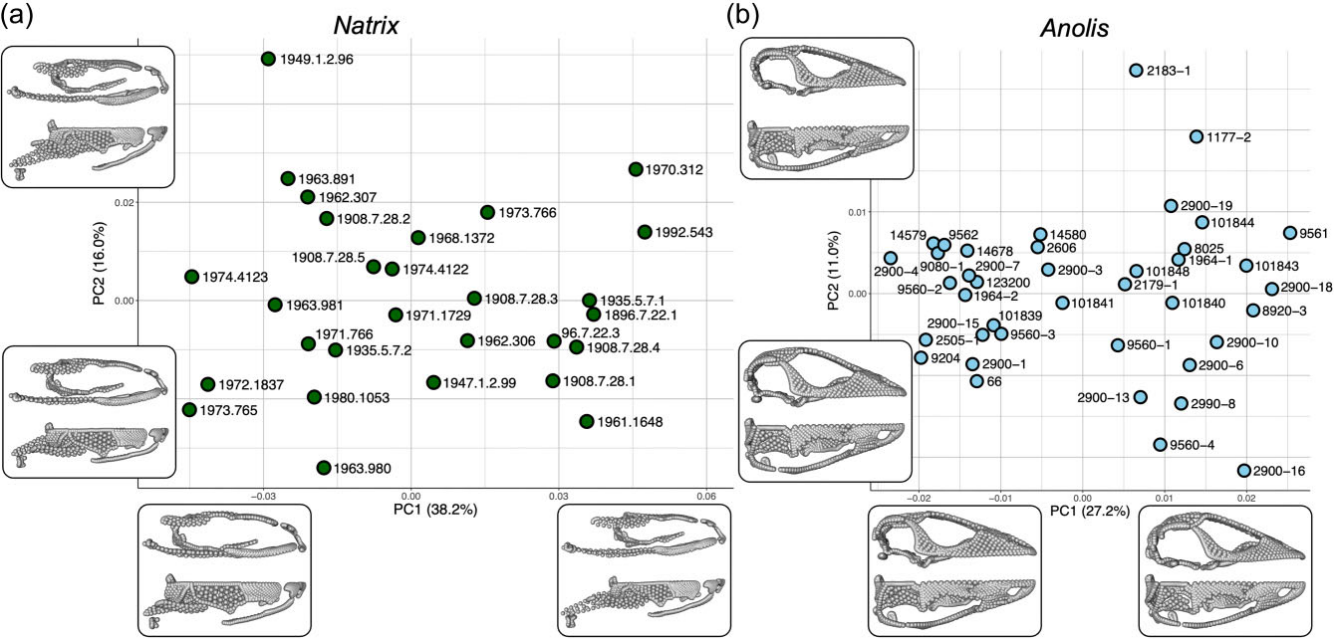
Morphospace constructed from first two PCs of skull shape for (a) *Natrix helvetica* and (b) *Anolis carolinensis* specimens. Labels indicate specimen numbers, where all *N. helvetica* specimens are from NHMUK (Natural History Museum, London) and *A. helvetica* specimens are from FLMNH (Florida Museum of Natural History). Inset images show extreme shape differences along PC axes in lateral (top) and dorsal (bottom) views.

PC analysis reveals that the first two PC axes together account for 54.2 and 38.2% of total skull shape variation in *N. helvetica* and *A. carolinensis*, respectively (Fig. 2). In *N. helvetica*, PC1 is associated with the anteroposterior extent of the neurocranium, with high variation in the relative position of the jaw joint. PC2 corresponds to the mediolateral position of the maxilla, pterygoid, and the jaw joint. In *A. carolinensis*, shape changes along PC1 comprises relative anteroposterior length of the rostrum and occiput region, mediolateral extent of basioccipital, and the anteroposterior position of the quadrate relative to the rest of the skull. PC2 broadly correlates with the relative length and depth of the skull.

### Allometry

Allometry is a significant factor in both species (*P* < 0.05). In *N. helvetica* and *A. carolinensis*, allometry accounts for 8.2 and 24.9% of their respective total skull shape variation.

### Intraspecific cranial integration

**Fig. 3.**
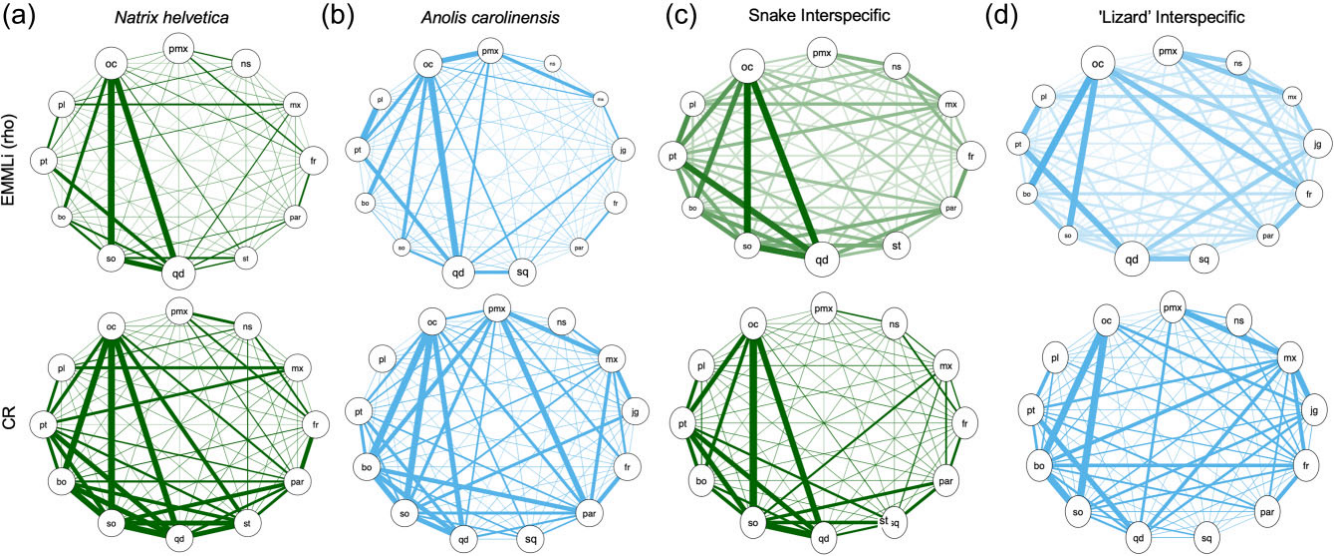
Network diagrams depicting the pattern of cranial integration in (a) *Natrix helvetica*, (b) *Anolis carolinensis*, (c) snakes, and (d) “lizards.” In the top row, the thickness of lines and diameter of circles indicate the strength of correlation (⍴) between and within partitions, respectively, from EMMLi analysis. In the bottom row, the thickness of lines is proportionate to CR values. Abbreviations: pmx, premaxilla; ns, nasal; jg, jugal; mx, maxilla; fr, frontal; par, parietal; sq, squamosal; st, supratemporal, qd, quadrate (jaw joint); so, supra-otoccipital; bo, basioccipital; pt, pterygoid; pl, palatine; oc, occipital condyle.

Based on CR and EMMLi analyses, *N. helvetica* and *A. carolinensis* exhibit shared as well as distinct patterns in cranial integration (Table 3; Fig. 3). In *N. helvetica*, we observe strong integration (in upper 20% of range in CR and ρ values) between the occipital elements (supra-otoccipital, basioccipital, and occipital condyle) and jaw joint on the quadrate bone. The frontal and parietal bones exhibit elevated CR and ρ values (CR = 0.80; ρ = 0.41). The jaw joint on the quadrate has the greatest within-region correlation (ρ = 0.98) of the cranial bones. The lowest within-region correlation value was demonstrated by the basioccipital (ρ = 0.60). With respect to CR and ρ values, the covariation between the adjacent regions supra-otoccipital and occipital condyle is the strongest (CR = 0.96; ρ = 0.77). Both the frontal-parietal and pterygoid-palatine pairs exhibit moderate level of integration (near the middle range of CR and ρ values). The relative strength of cranial integration among partitions in the posterior skull is generally reduced in the EMMLi analysis, however the occipital condyle remains a major conduit in the posterior skull. In the viscerocranium, relatively strong integration is found between premaxilla-frontal (CR = 0.66; ρ = 0.39) and premaxilla-nasal (CR = 0.72; ρ = 0.37).

**Table 3.** Between-partition covariance ratios (upper triangle), between-partition correlations (bottom triangle), and within-partition correlations (diagonal) across cranial regions. Values calculated using *modularity.test* in the *geomorph* R package and EMMLi, respectively. Greater ρ and CR values denote greater level of integration between pairs of elements. Darker shades of grey on off-diagonal elements denote relatively stronger integration, where five degrees of shades were applied based on range of ρ and CR values divided into five equal bins.

*Natrix helvetica*
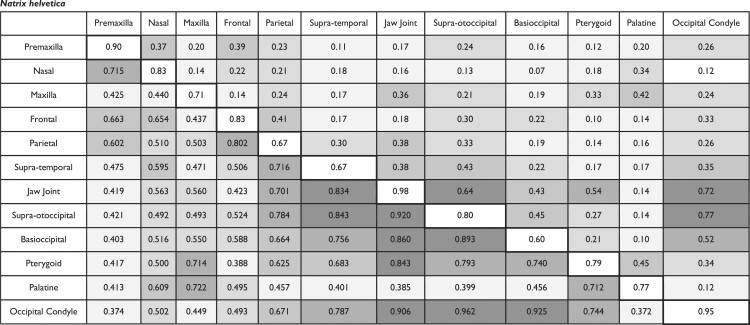
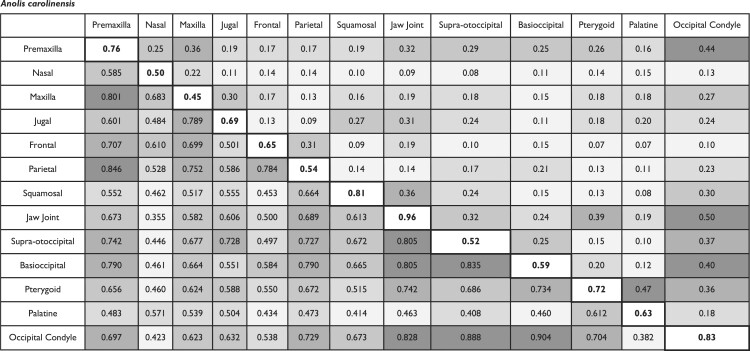

Similar to *N. helvetica, A. carolinensis* also shows strong between-region integration among occipital and palate elements (Table 3; Fig. 3). For example, the occipital condyle and the basioccipital show the highest CR value for between-region integration, with a relatively high ρ value (CR = 0.90; ρ = 0.40). As in *N. helvetica*, the EMMLi analysis shows the greatest within-region integration for the jaw joint on the quadrate (ρ = 0.96). Likewise, *A. carolinensis* shares moderate to relatively strong integration between the frontal and parietal bones (CR = 0.784 in ≥60 percentile of the range in value; ρ = 0.31 in >40 percentile). In contrast to *N. helvetica, A. carolinensis* shows moderate to relatively strong integration between the rostral bones, including the premaxilla and maxilla (CR = 0.80 in ≥80 percentile; ρ = 0.36 in >60 percentile), and between the pterygoid and palatine bones based on the value (ρ = 0.47; >80 percentile). Taken together, *A. carolinensis* displays elevated levels of integration between the premaxilla and maxilla; frontal and parietal; pterygoid and palatine; and occipital regions (supra-otoccipital, basioccipital, and occipital condyle). In addition, jaw joint and occipital condyle as well as pterygoid and jaw joint exhibit heightened degree of integration as in *N. helvetica*.

### Comparison to interspecific integration pattern

The comparison of the intraspecific pattern of integration in *N. helvetica* to the results of a phylogenetically corrected snake-wide analysis (Watanabe et al. 2019) shows key similarities. Notably, the presence of moderate to strongly integrated “occiput” and “palate” regions, with modular viscerocranium is common between the *N. helvetica* and snake-wide datasets. In addition, the frontal and parietal bones show relatively strong between-region integration in *N. helvetica* (CR = 0.80; ρ = 0.41) and the interspecific data (CR = 0.60; ρ = 0.39). Furthermore, the interspecific (snake-wide) data showed the jaw joint on the quadrate to have strong within-region integration (ρ = 0.99) and the basioccipital to have the lowest within-region integration (ρ = 0.61). This outcome is replicated in *N. helvetica*, where the jaw joint on the quadrate has the highest within-region integration (ρ = 0.98) and the basioccipital has the lowest within-region integration (ρ = 0.60).

When compared to the interspecific lizard data, the pattern of cranial integration within *A. carolinensis* is largely congruent, with some exceptions. Notably, the intraspecific pattern shares moderately to strongly integrated “rostrum,” “skull roof,” “palate,” and “occiput” regions that were reported for lizard-wide analysis of cranial integration (Watanabe et al. 2019). Between-region integration from the lizard interspecific dataset and *A. carolinensis* data indicate weak integration between the premaxilla-squamosal, nasal-occipital condyle, frontal-squamosal, frontal-palatine, and squamosal-palatine bones. The maxilla (ρ = 0.54) and supra-otoccipital (ρ = 0.54) present the lowest within-region integration for the lizard-wide dataset. *Anolis carolinensis* exhibits similar within-region integration for the maxilla (ρ = 0.45) and supra-otoccipital (ρ = 0.52), with the nasal bone also showing weak within-region integration (ρ = 0.50). The jaw joint on the quadrate shows the strongest within-region integration interspecifically (ρ = 0.98) and intraspecifically (ρ = 0.96). Meanwhile, we observe relatively weak integration between the occipital condyle and the jaw joint (quadrate) across “lizards” that is not reflected in the intraspecific *A. carolinensis* analysis. The *A. carolinensis* specimens exhibit a strong integrating signal between the occipital condyle and the quadrate (CR = 0.83; ρ = 0.50; >80 percentile), whereas the interspecific lizard data have a relatively low level of integration (CR = 0.689; ρ = 0.36).

**Fig 4.**
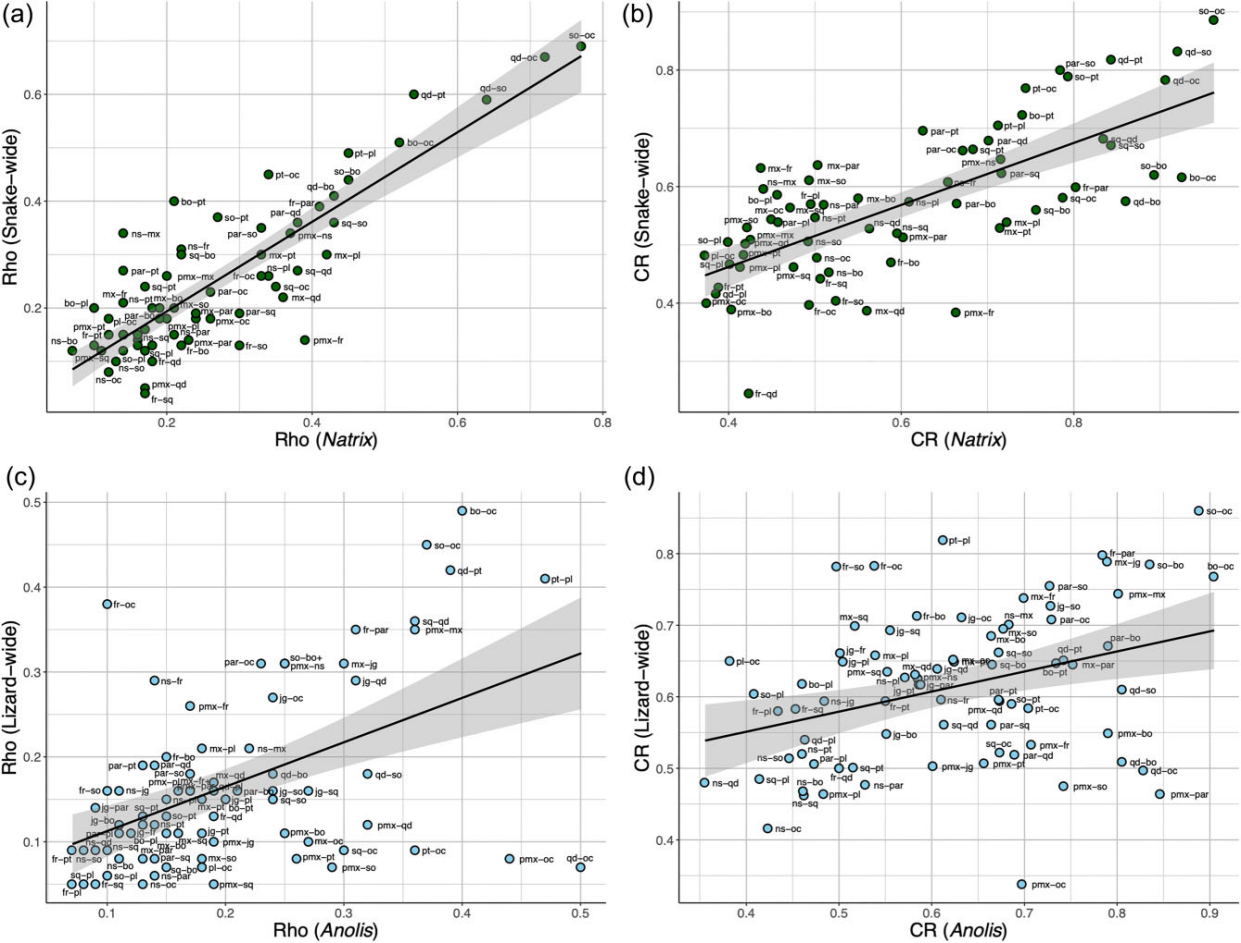
Bivariate plots of between-region integration of interspecific against intraspecific data. (a) ⍴ values for snake-wide and *Natrix* data; (b) CR values for snake-wide and *Natrix* data; (c) ⍴ values for lizard-wide and *Anolis* data; and (d) CR values for lizard-wide and *Anolis* data. Black lines and grey bands indicate best fit regression lines and 95% confidence intervals, respectively. Text labels denote the pair of regions associated with the integration value (some labels in dense clusters of points were removed to improve legibility). Abbreviations: pmx, premaxilla; ns, nasal; jg, jugal; mx, maxilla; fr, frontal; par, parietal; sq, squamosal; st, supratemporal, qd, quadrate (jaw joint); so, supra-otoccipital; bo, basioccipital; pt, pterygoid; pl, palatine; oc, occipital condyle.

Quantitative comparison of intra- and interspecific patterns in cranial integration supports the results described above and provides new insights. First, correlation analysis on CR and ρ values show that there is a broad correspondence in between-region integration when comparing intra- and interspecific datasets. While deviations from the best fit regression line are apparent (Fig. 4), the relative degree of integration is strongly correlated between *N. helvetica* and snake-wide datasets (*R*^2^ρ = 0.723, *P* < 0.0001; *R*^2^_CR_ = 0.512, *P* < 0.0001). Likewise, the correlation between *A. carolinensis* and lizard-wide dataset with respect to CR and ρ values is statistically significant (*R*^2^ρ = 0.243, *P* < 0.0001; *R*^2^_CR_ = 0.122, *P* = 0.0017). As reflected in the results of the correlation analysis, the bivariate plots of intra- and interspecific integration (Fig. 4) depict tighter correspondence in CR and ρ values for *N. helvetica* and snake-wide datasets, compared to those of *A. carolinensis* and lizard-wide data. Moreover, the plots reveal pairs of skull regions that deviate from the association between intra- and interspecific patterns. For example, *N. helvetica* exhibits greater ρ values for premaxilla-frontal integration than the snake-wide pattern, whereas snakes show relatively stronger nasal-maxilla, frontal-quadrate, and basioccipital-pterygoid integration than *N. helvetica* (Fig. 4a, b). Among lizards, *A. carolinensis* presents stronger degree of integration between the occipital condyle with the premaxilla, jaw joint, and pterygoid than in lizard-wide data, whereas frontal-occipital, pterygoid-palatine, and supra-otoccipital-occipital condyle integration is relatively stronger across lizards than within *A. carolinensis* (Fig. 4c, d).

## Discussion

### Intraspecific cranial integration

Compared to a previous study on cranial integration in *N. natrix* and *N. tessellata* (Andjelković et al. 2017), our results for *N. helvetica* show both similarities and differences. For the regions characterized here, the previously published dataset also showed statistically significant covariations between the braincase (partitioned as single anatomical unit) and quadrate, as well as a strong association between the maxilla and palatine bones. The maxilla, pterygoid, and the jaw joint are highly mobile elements in snake skulls. As such, their functional properties may underlie the strong integration patterns across these regions. For instance, PC2 axis is associated with the relative position of these mobile elements, suggesting that these regions undergo coordinated changes in shape and relative position. The more moderate integration between the palatine and pterygoid in *Natrix* and snake-wide data, as replicated in our dataset, could be due to the low contact point between these two bones in snakes. In contrast to Andjelković et al.’s (2017), results from other *Natrix* species, our *N. helvetica* data lack evidence of strong integration between the maxilla, quadrate, and supratemporal elements. This result could represent genuine differences in cranial integration between *Natrix* species. However, these discrepancies could also be due to the differing landmarking approach and metrics for evaluating phenotypic integration. Andjelković and colleagues employed only fixed landmarks when characterizing skull shape and identified integrated pairs of regions based on statistical significance using the RV coefficient (Escoufier 1973; Klingenberg 2013). Results of integration analysis are known to be influenced by the type of landmarks (e.g., inclusion of semi-landmarks), number of landmarks, and specimen sampling (e.g., Adams 2016; Cardini, 2019). However, the general principle that separated elements of the rostrum and palate in snakes are more weakly integrated than more connected elements, such as the neurocranium, remains true across both studies.

The integration pattern found here for *A. carolinensis* also shows a mixture of congruent and contrasting results with a previous comprehensive study on cranial integration in *Anolis* species (Sanger et al. 2012). Using two-dimensional geometric morphometric data, Sanger and colleagues (2012) found that the “tripartite” model of integration is supported for *A. carolinensis*, where the rostrum, orbit, and braincase form integrated regions. Although the difference in landmarking scheme and sampling preclude direct comparison of results, the integration patterns observed are generally consistent. Although our results lack evidence for an “orbit” module, the braincase, including the occiput, and the rostrum are strongly integrated. It is worth noting that our *A. carolinensis* sampling consists predominantly of individuals from Alachua County in Florida, with the exception of two specimens (FLMNH 2990–8,101848). While there is a possibility that these two individuals may be members of populations with different cranial integration patterns, the morphospace (Fig. 2b) shows that their skull shapes are well within the variation exhibited by other specimens. Therefore, we consider the cranial integration pattern presented here for *A. carolinensis* to be a reliable, at least for the pattern exhibited by populations in Alachua County.

### Comparison with interspecific integration

The intraspecific patterns of cranial integration within *A. carolinensis* and *N. helvetica* resemble the interspecific patterns observed across lizards and snakes, respectively. These two distinct levels of analyses present a shared set of highly integrated regions, including the “skull roof” (frontal, nasal), “palate” (pterygoid, palatine), and “occiput” (supra-otoccipital, basioccipital, and occipital condyle). Moreover, *A. carolinensis* and lizard-wide analyses show a more integrated rostrum than *N. helvetica* and other snakes. In contrast, *N. helvetica* specimens have a more modular rostral region, in agreement with the interspecific snake-wide pattern (Watanabe et al. 2019). This difference in the rostral region between snakes and lizards could be related to the level of articulation among rostral elements. In snakes, rostral elements, including the premaxilla, nasal, and maxilla bones, do not closely articulate with one another as in lizards.

The overall congruence in integration patterns of *N. helvetica* and *A. carolinensis* to the snake- and lizard-wide analyses is notable. This outcome suggests that the processes that shape trait covariation at the population level extends to large-scale evolutionary processes. Previous studies that have examined intraspecific and interspecific patterns of integration and modularity generally corroborate this finding. For instance, the skull of ring-necked parakeet supports the same nine-module hypothesis observed across crown birds (Mitchell et al. 2021), and interspecific and static patterns of cranial integration are similar in caecilians (Marshall et al. 2019). In lacertids, datasets capturing static and interspecific variation supported different integration hypotheses (“anteroposterior” and “neurodermatocranial”, respectively; Urošević et al. 2019). However, these modularity hypotheses are quite similar in the partitioning of landmarks into units, implying that intra- and interspecific patterns of cranial integration match to a large extent.

Consequently, phenotypic covariation within species could be used to predict covariation across clades, and vice versa, thereby unifying micro- and macroevolutionary dynamics. This conclusion that integration pattern and modules are conserved across multiple hierarchical levels seemingly contradicts the observation that pattern of modularity changes with cranial shape across *Anolis* species, potentially due to differences in diet (Butler & Losos 2002; Sanger et al. 2012). However, these contrasting implications are not necessarily incompatible. We acknowledge and fully expect that the pattern and strength of phenotypic covariation evolves along and differ across lineages. In fact, there are differences in intraspecific and interspecific patterns of cranial integration, as noted above, that could signify how the sampled species have deviated from the overall clade-wide patterns. Importantly, this study demonstrates that the strongly integrated elements are broadly maintained across micro- and macroevolutionary timelines, with limited number of exceptions (e.g., highly integrated quadrate-occipital in *A. carolinensis* that is absent in lizard-wide pattern). Individual lineages are still able to modify the degree of integration from this conserved pattern.

### Allometry and sexual dimorphism

Allometry is often a strong integrating force in biological structures (Zelditch & Fink 1995; Klingenberg 2013; Bright et al. 2016). Both *N. helvetica* and *A. carolinensis* show statistically significant allometric signal in their skull shapes, with greater effect of size in the latter. Although some studies on modularity and integration have performed analyses on allometry-corrected data (Sanger et al. 2012; Urošević et al. 2019), we found that analysis of allometry-corrected data strengthens the relative covariation and correlation of other pairs of elements (Fig. S1). While these results may include biologically informative information, the greater number of similarly integrated pairs of elements suggests that removing the overall allometric signal, known as a strong integrating factor, may dilute the original integration signal (Zeldith & Fink 1995; Klingenberg 2009; but see Urošević et al. 2020). In addition, keeping the allometric signal maintains the equivalency of our intraspecific analysis with the squamate-wide analysis. Therefore, we did not focus on reporting the results from the allometry-corrected analysis.

**Fig. 5.**
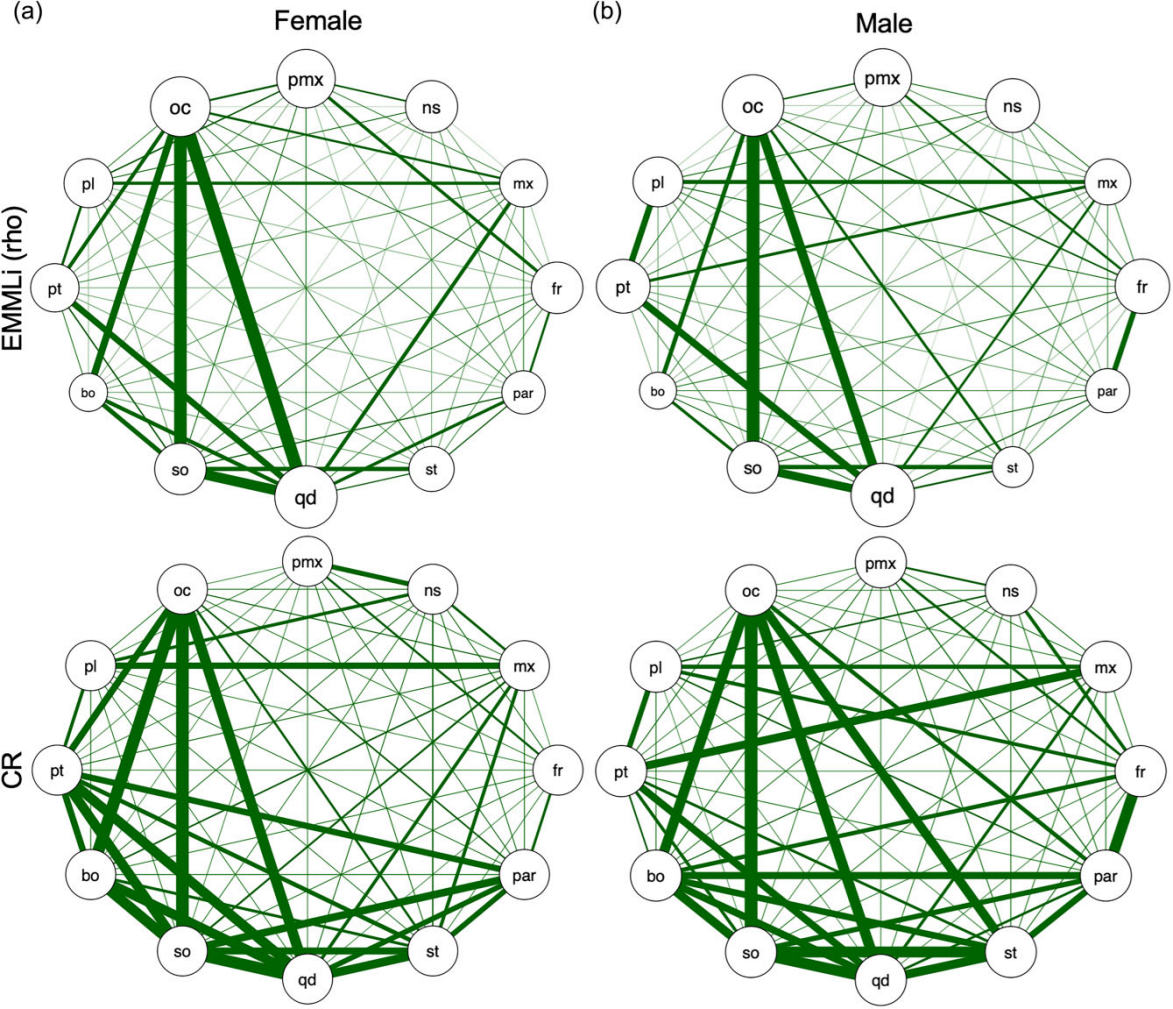
Network diagrams depicting the pattern of cranial integration in (a) female and (b) male specimens of *Natrix helvetica*. In the top row, the thickness of lines and diameter of circles indicate the strength of correlation (⍴) between-regions and within-regions, respectively, from EMMLi analysis. In the bottom row, the thickness of lines is proportionate to CR values. Abbreviations: pmx, premaxilla; ns, nasal; jg, jugal; mx, maxilla; fr, frontal; par, parietal; sq, squamosal; st, supratemporal, qd, quadrate (jaw joint); so, supra-otoccipital; bo, basioccipital; pt, pterygoid; pl, palatine; oc, occipital condyle.

Sexual dimorphism is known to occur in species of *Natrix* (Andjelković et al. 2016) and *Anolis* (Schoener 1967; Losos 2011). Ecological diversity is produced by sexual differences in *Anolis* species as males are typically seen on larger and higher perches compared to females and due to differences in the ecological niche of males and females (Schoener 1967; Butler et al. 2007). This difference in size across species and between sexes may contribute to changes in modularity and integration regarding functional aspects such as locomotion and feeding. Unfortunately, we lacked sex information on sampled *A. carolinensis* specimens, preventing a proper assessment of sex-related differences in shape and integration. While we do not see a visual indication of cranial shape dimorphism in the morphospace for *A. carolinensis* (Fig. 2), allometry accounts for nearly a quarter of the skull shape variation. As such, known sexual size dimorphism in *Anolis* is expected to be associated with skull shape differences, which has been demonstrated previously (Herrel et al. 2007). Whether this shape difference would lead to divergence in integration patterns is unclear. For *N. helvetica*, which sex information was known (Table 1), we observed weak support for sexual shape dimorphism in the skull (*R*^2^ = 0.082; *P* = 0.041). This result counters a previous study showing lack of sexual shape dimorphism in the dorsal aspect of the head in *N. helvetica* (Tamagnini et al. 2018), but is concordant with known sexual shape dimorphism reported for *N. natrix* and *N. tessellata*, where different sets of bones were sexually dimorphic between these two species (Andjelković et al. 2016). Despite the presence of sexual shape dimorphism, separate CR and EMMLi analyses on female and male datasets demonstrate equivalent patterns, with the exception of stronger relative integration between the frontal and parietal bones in males (Fig. 5; Table S1). While there may be subtle differences in cranial integration between sexes (e.g., relatively more integrated palatine-pterygoid and frontal-parietal in males), the results and conclusions of this study remain broadly consistent.

## Conclusions

In this study, we use a high-density geometric morphometric approach to document the pattern of cranial integration in *N. helvetica* and *A. carolinensis*. We then compare these intraspecific patterns to snake- and lizard-wide cranial integration, respectively, from a previous study using a comparable landmarking scheme. We find that intraspecific patterns of cranial integration in *N. helvetica* and *A. carolinensis* species share the following integrated regions: skull roof (frontal, parietal), occiput (supra-otoccipital-basioccipital-occipital condyle-quadrate), and palate (palatine-pterygoid). *Anolis carolinensis* shows a more integrated rostrum (premaxilla-nasal-maxilla) than in *N. helvetica* specimens. Overall, the intraspecific patterns of covariation in *N. helvetica* and *A. carolinensis* generally match the snake- and lizard-wide interspecific patterns, respectively, with few exceptions that likely represent lineage-specific deviations from clade-wide trends. Therefore, cranial integration within species may direct clade-wide morphological evolution to a large and detectable degree; thus, unifying micro- and macroevolutionary trends, and potentially process, in phenotypic evolution.

## Supplementary Material

obad022_Supplemental_Files

## Data Availability

The data and code used in this study are in the Supplementary Information.

## References

[bib1] Adams DC . 2016. Evaluating modularity in morphometric data: challenges with the RV coefficient and a new test measure. Methods Ecol Evol, 7:565–72.

[bib2] Adams DC , Otárola-Castillo E. 2013. geomorph: an R package for the collection and analysis of geometric morphometric shape data. Methods Ecol Evol, 4:393–9.

[bib3] Andjelković M , Tomović L, Ivanović A. 2016. Variation in skull size and shape of two snake species (*Natrix natrix* and *Natrix tessellata*). Zoomorphology, 135:243–53.

[bib4] Andjelković M , Tomović L, Ivanović A. 2017. Morphological integration of the kinetic skull in *Natrix* snakes. J Zool, 303:188–98.

[bib5] Bardua C , Fabre AC, Bon M, Das K, Stanley EL, Blackburn DC, Goswami A. 2020. Evolutionary integration of the frog cranium. Evolution, 74:1200–15. 32346857 10.1111/evo.13984

[bib6] Bardua C , Felice RN, Watanabe A, Fabre A-C, Goswami A. 2019. A practical guide to sliding and surface semilandmarks in morphometric analyses. Integr Org Biol, 1:obz016. 33791531 10.1093/iob/obz016 PMC7780474

[bib7] Botton-Divet L , Cornette R, Fabre A-C, Herrel A, Houssaye A. 2016. Morphological analysis of long bones in semi-aquatic mustelids and their terrestrial relatives. Integr Comp Biol, 56:1298–309. 27794537 10.1093/icb/icw124

[bib8] Bright JA , Marugán-Lobón J, Cobb SN, Rayfield EJ. 2016. The shapes of bird beaks are highly controlled by nondietary factors. Proc Natl Acad Sci, 113:5352–7. 27125856 10.1073/pnas.1602683113 PMC4868483

[bib9] Butler MA , Losos JB. 2002. Multivariate sexual dimorphism, sexual selection, and adaptation in Greater Antillean *Anolis* lizards. Ecol Monogr, 72:541–59.

[bib10] Butler MA , Sawyer SA, Losos JB. 2007. Sexual dimorphism and adaptive radiation in *Anolis* lizards. Nature, 447:202–5. 17495925 10.1038/nature05774

[bib11] Cardini A . 2016. Lost in the other half: improving accuracy in geometric morphometric analyses of one side of bilaterally symmetric structures. Syst Biol, 65:1096–106. 27288476 10.1093/sysbio/syw043

[bib13] Cardini A . 2017. Left, right or both? estimating and improving accuracy of one-side-only geometric morphometric analyses of cranial variation. J Zool Syst Evol Res, 55:1–10.

[bib12] Cardini A . 2019. Integration and modularity in Procrustes shape data: is there a risk of spurious results? Evol Biol 46:90–105.

[bib14] Escoufier Y . 1973. Le traitement des variables vectorielles. Biometrics, 29:751–60.

[bib15] Evans KM , Buser TJ, Larouche O, Kolmann MA. 2022. Untangling the relationship between developmental and evolutionary integration. Semin Cell Dev Biol, 145:22–27. 35659472 10.1016/j.semcdb.2022.05.026

[bib16] Fabre AC , Bardua C, Bon M, Clavel J, Felice RN, Streicher JW, Bonnel J, Stanley EL, Blackburn DC, Goswami A. 2020. Metamorphosis shapes cranial diversity and rate of evolution in salamanders. Nat Ecol Evol, 4:1129–40. 32572219 10.1038/s41559-020-1225-3

[bib17] Felice RN , Goswami A. 2018. Developmental origins of mosaic evolution in the avian cranium. Proc Natl Acad Sci, 115:555–60. 29279399 10.1073/pnas.1716437115 PMC5776993

[bib18] Felice RN , Randau M, Goswami A. 2018. A fly in a tube: macroevolutionary expectations for integrated phenotypes. Evolution, 72:2580–94. 30246245 10.1111/evo.13608 PMC6585935

[bib19] Felice RN , Watanabe A, Cuff AR, Hanson M, Bhullar BAS, Rayfield ER, Witmer LM, Norell MA, Goswami A. 2020. Decelerated dinosaur skull evolution with the origin of birds. PLoS Biol, 18:e3000801. 32810126 10.1371/journal.pbio.3000801 PMC7437466

[bib20] Fritz U , Schmidtler JF. 2020. The fifth labour of Heracles: cleaning the linnean stable of names for grass snakes (*Natrix astreptophora, N. helvetica, N. natrix* sensu stricto). Vertebr Zool, 70:621–65.

[bib21] Goswami A , Finarelli JA. 2016. EMMLi: a maximum likelihood approach to the analysis of modularity. Evolution, 70:1622–37. 27188434 10.1111/evo.12956

[bib22] Goswami A , Smaers JB, Soligo C, Polly PD. 2014. The macroevolutionary consequences of phenotypic integration: from development to deep time. Philos Trans R Soc B Biol Sci, 369:20130254. 10.1098/rstb.2013.0254 PMC4084539 25002699

[bib23] Gunz P , Mitteroecker P. 2013. Semilandmarks: a method for quantifying curves and surfaces. Hystrix Ital J Mammal, 24:103–9.

[bib24] Gunz P , Mitteroecker P, Bookstein FL. 2005. Semilandmarks in three dimensions. In: Modern morphometrics in Physical Anthropology. New York: Springer. p. 73–98.

[bib25] Hallgrímsson B , Jamniczky H, Young NM, Rolian C, Parsons TE, Boughner JC, Marcucio RS. 2009. Deciphering the palimpsest: studying the relationship between morphological integration and phenotypic covariation. Evol Biol, 36:355–76. 23293400 10.1007/s11692-009-9076-5 PMC3537827

[bib26] Herrel A , McBrayer LD, Larson PM. 2007. Functional basis for sexual differences in bite force in the lizard *Anolis carolinensis*. Biol J Linn Soc Lond, 91:111–9.

[bib27] Klingenberg CP . 2008. Morphological integration and developmental modularity. Annu Rev Ecol Evol Syst, 39:115–32.

[bib28] Klingenberg CP . 2009. Morphometric integration and modularity in configurations of landmarks: tools for evaluating a-priori hypotheses. Evol Dev, 11:405–21. 19601974 10.1111/j.1525-142X.2009.00347.x PMC2776930

[bib29] Klingenberg CP . 2013. Cranial integration and modularity: insights into evolution and development from morphometric data. Hystrix Ital. J. Mammal, 24:43–58.

[bib30] Klingenberg CP . 2014. Studying morphological integration and modularity at multiple levels: concepts and analysis. Philos Trans R Soc B Biol Sci, 369:33–5. 10.1098/rstb.2013.0249 PMC4084535 25002695

[bib31] Losos JB . 2011. Lizards in an evolutionary tree: Ecology and adaptive radiation of anoles. Oakland: University of California Press.

[bib32] Marshall AF , Bardua C, Gower DJ, Wilkinson M, Sherratt E, Goswami A. 2019. High-density three-dimensional morphometric analyses support conserved static (intraspecific) modularity in caecilian (Amphibia: Gymnophiona) crania. Biol J Linn Soc, 126:721–42.

[bib33] Mitchell MJ , Goswami A, Felice RN. 2021. Cranial integration in the ring-necked parakeet, *Psittacula krameri* (Psittaciformes: Psittaculidae). Biol J Linn Soc, 133:47–56.

[bib34] Mitteroecker P . 2009. The developmental basis of variational modularity: insights from quantitative genetics, morphometrics, and developmental biology. Evol Biol, 36:377–85.

[bib35] Olson EC , Miller RL. 1958. Morphological integration. Chicago: University of Chicago Press, (reprinted 1999).

[bib36] Porto A , de Oliveira FB, Shirai LT, de Conto V, Marroig G. 2009. The evolution of modularity in the mammalian skull I: morphological integration patterns and magnitudes. Evolutionary Biology, 36:118–35.

[bib37] R Core Development Team . 2022. R: A language and environment for statistical computing. R Foundation for Statistical Computing. https://www.r-project.org

[bib38] RStudio Team . 2020. RStudio: Integrated Development for R. Boston, MA: RStudio, PBC. URL http://www.rstudio.com/.

[bib39] Sanger TJ , Mahler DL, Abzhanov A, Losos JB. 2012. Roles for modularity and constraint in the evolution of cranial diversity among *Anolis* lizards. Evol Int J Org Evol, 66:1525–42. 10.1111/j.1558-5646.2011.01519.x 22519788

[bib40] Schlager S . 2017. Morpho and Rvcg–shape analysis in R: R-packages for geometric morphometrics, shape analysis and surface manipulations. In: Statistical Shape and Deformation Analysis. London: Elsevier. p. 217–56.

[bib41] Schoener TW . 1967. The ecological significance of sexual dimorphism in size in the lizard *Anolis conspersus*. Science, 155:474–7. 17737565 10.1126/science.155.3761.474

[bib42] Tamagnini D , Stephenson J, Brown RP, Meloro C. 2018. Geometric morphometric analyses of sexual dimorphism and allometry in two sympatric snakes: *Natrix helvetica* (Natricidae) and *Vipera berus* (Viperidae). Zoology, 129:25–34. 30170745 10.1016/j.zool.2018.05.008

[bib43] Tollis M , Ausubel G, Ghimire D, Boissinot S. 2012. Multi-locus phylogeographic and population genetic analysis of *Anolis carolinensis*: historical demography of a genomic model species. PLoS One 7:e38474. 22685573 10.1371/journal.pone.0038474 PMC3369884

[bib44] Urošević A , Ajduković M, Arntzen JW, Ivanović A. 2020. Morphological integration and serial homology: a case study of the cranium and anterior vertebrae in salamanders. J Zool Syst Evol Res, 58:1206–19.

[bib45] Urošević A , Ljubisavljević K, Ivanović A. 2019. Multilevel assessment of the Lacertid lizard cranial modularity. J Zoolog Syst Evol Res, 57:145–58.

[bib46] Wagner GP . 1996. Homologues, natural kinds and the evolution of modularity. Am Zool, 36:36–43.

[bib47] Wagner GP , Altenberg L. 1996. Perspective: Complex adaptations and the evolution of evolvability. Evolution, 50:967–76. 28565291 10.1111/j.1558-5646.1996.tb02339.x

[bib48] Watanabe A , Fabre A-C, Felice RN, Maisano JA, Müller J, Herrel A, Goswami A. 2019. Ecomorphological diversification in squamates from conserved pattern of cranial integration. Proc Natl Acad Sci, 116:14688–97. 31262818 10.1073/pnas.1820967116 PMC6642379

[bib49] Wiley DF , Amenta N, Alcantara DA, Ghosh D, Kil YJ, Delson E, Harcourt-Smith W, Rohlf FJ, St John K, Hamann B. 2005. Evolutionary morphing. IEEE Visualization, 2005:431–438.

[bib50] Zelditch ML , Fink WL. 1995. Allometry and developmental integration of body growth in a piranha, *Pygocentrus nattereri* (Teleostei: Ostariophysi. J Morphol, 223:341–55. 29865299 10.1002/jmor.1052230309

