# Supplementary material for: High-Density Geometric Morphometric Analysis of Intraspecific Cranial Integration in the Barred Grass Snake (Natrix helvetica) and Green Anole (Anolis carolinensis)
Source: Integr Org Biol. 2023 Jun 5;5(1):obad022. doi: 10.1093/iob/obad022 (PMC10311474; doi:10.1093/iob/obad022)
Supplement: obad022_Supplemental_Files [file obad022_supplemental_files.zip › TableS1.docx]

**Table S1 Between-partition covariance ratios (upper triangle), between-partition correlations (bottom triangle), and within-partition correlations (diagonal) across cranial regions.** Values calculated using *modularity.test* in the *geomorph* R package and EMMLi, respectively. Greater ρ and CR values denote greater level of integration between pairs of elements.

*Natrix helvetica* (Female)

|  | Premaxilla | Nasal | Maxilla | Frontal | Parietal | Supratemporal | Quadrate | Supra-otoccipital | Basioccipital | Pterygoid | Palatine | Occipital Condyle |
| --- | --- | --- | --- | --- | --- | --- | --- | --- | --- | --- | --- | --- |
| Premaxilla | 0.92 | 0.37 | 0.26 | 0.42 | 0.22 | 0.14 | 0.31 | 0.31 | 0.17 | 0.21 | 0.36 | 0.38 |
| Nasal | 0.774 | 0.82 | 0.09 | 0.23 | 0.23 | 0.17 | 0.13 | 0.09 | 0.09 | 0.14 | 0.33 | 0.07 |
| Maxilla | 0.545 | 0.719 | 0.75 | 0.14 | 0.29 | 0.21 | 0.44 | 0.30 | 0.22 | 0.25 | 0.42 | 0.39 |
| Frontal | 0.711 | 0.673 | 0.470 | 0.81 | 0.39 | 0.24 | 0.26 | 0.31 | 0.21 | 0.17 | 0.16 | 0.33 |
| Parietal | 0.677 | 0.663 | 0.655 | 0.722 | 0.68 | 0.35 | 0.41 | 0.35 | 0.18 | 0.17 | 0.13 | 0.28 |
| Supratemporal | 0.663 | 0.692 | 0.740 | 0.531 | 0.784 | 0.71 | 0.35 | 0.46 | 0.20 | 0.19 | 0.22 | 0.31 |
| Quadrate | 0.530 | 0.676 | 0.737 | 0.478 | 0.783 | 0.859 | 0.98 | 0.67 | 0.45 | 0.54 | 0.18 | 0.76 |
| Supra-otoccipital | 0.494 | 0.565 | 0.706 | 0.609 | 0.836 | 0.822 | 0.931 | 0.81 | 0.47 | 0.36 | 0.22 | 0.76 |
| Basioccipital | 0.445 | 0.624 | 0.698 | 0.596 | 0.660 | 0.727 | 0.880 | 0.904 | 0.60 | 0.29 | 0.13 | 0.56 |
| Pterygoid | 0.465 | 0.545 | 0.671 | 0.481 | 0.805 | 0.761 | 0.891 | 0.887 | 0.811 | 0.76 | 0.40 | 0.44 |
| Palatine | 0.523 | 0.737 | 0.806 | 0.507 | 0.616 | 0.591 | 0.570 | 0.591 | 0.590 | 0.737 | 0.77 | 0.26 |
| Occipital Condyle | 0.465 | 0.582 | 0.684 | 0.554 | 0.699 | 0.715 | 0.901 | 0.952 | 0.938 | 0.815 | 0.573 | 0.94 |

*Natrix helvetica* (Male)

|  | Premaxilla | Nasal | Maxilla | Frontal | Parietal | Supra-temporal | Quadrate | Supra-otoccipital | Basi-occipital | Pterygoid | Palatine | Occipital Condyle |
| --- | --- | --- | --- | --- | --- | --- | --- | --- | --- | --- | --- | --- |
| Premaxilla | 0.89 | 0.30 | 0.21 | 0.38 | 0.27 | 0.12 | 0.20 | 0.27 | 0.24 | 0.10 | 0.12 | 0.35 |
| Nasal | 0.662 | 0.83 | 0.30 | 0.23 | 0.15 | 0.17 | 0.07 | 0.11 | 0.13 | 0.28 | 0.31 | 0.10 |
| Maxilla | 0.555 | 0.594 | 0.71 | 0.26 | 0.24 | 0.21 | 0.38 | 0.19 | 0.21 | 0.40 | 0.44 | 0.19 |
| Frontal | 0.676 | 0.700 | 0.572 | 0.84 | 0.49 | 0.15 | 0.11 | 0.29 | 0.27 | 0.20 | 0.32 | 0.35 |
| Parietal | 0.633 | 0.571 | 0.584 | 0.916 | 0.68 | 0.29 | 0.33 | 0.32 | 0.24 | 0.19 | 0.27 | 0.28 |
| Supratemporal | 0.616 | 0.606 | 0.556 | 0.593 | 0.790 | 0.63 | 0.36 | 0.45 | 0.22 | 0.23 | 0.23 | 0.39 |
| Quadrate | 0.483 | 0.551 | 0.689 | 0.448 | 0.673 | 0.852 | 0.98 | 0.60 | 0.32 | 0.55 | 0.19 | 0.64 |
| Supra-otoccipital | 0.519 | 0.475 | 0.548 | 0.497 | 0.751 | 0.911 | 0.914 | 0.80 | 0.40 | 0.23 | 0.13 | 0.77 |
| Basioccipital | 0.607 | 0.530 | 0.634 | 0.724 | 0.805 | 0.789 | 0.807 | 0.854 | 0.57 | 0.16 | 0.18 | 0.45 |
| Pterygoid | 0.455 | 0.607 | 0.832 | 0.453 | 0.541 | 0.681 | 0.811 | 0.693 | 0.653 | 0.83 | 0.52 | 0.27 |
| Palatine | 0.396 | 0.651 | 0.721 | 0.707 | 0.648 | 0.473 | 0.452 | 0.419 | 0.571 | 0.759 | 0.77 | 0.10 |
| Occipital Condyle | 0.503 | 0.445 | 0.503 | 0.517 | 0.721 | 0.863 | 0.903 | 0.970 | 0.889 | 0.623 | 0.415 | 0.95 |
