# Supplementary figures and images for: High-Density Geometric Morphometric Analysis of Intraspecific Cranial Integration in the Barred Grass Snake (Natrix helvetica) and Green Anole (Anolis carolinensis)
Source: Integr Org Biol. 2023 Jun 5;5(1):obad022. doi: 10.1093/iob/obad022 (PMC10311474; doi:10.1093/iob/obad022)

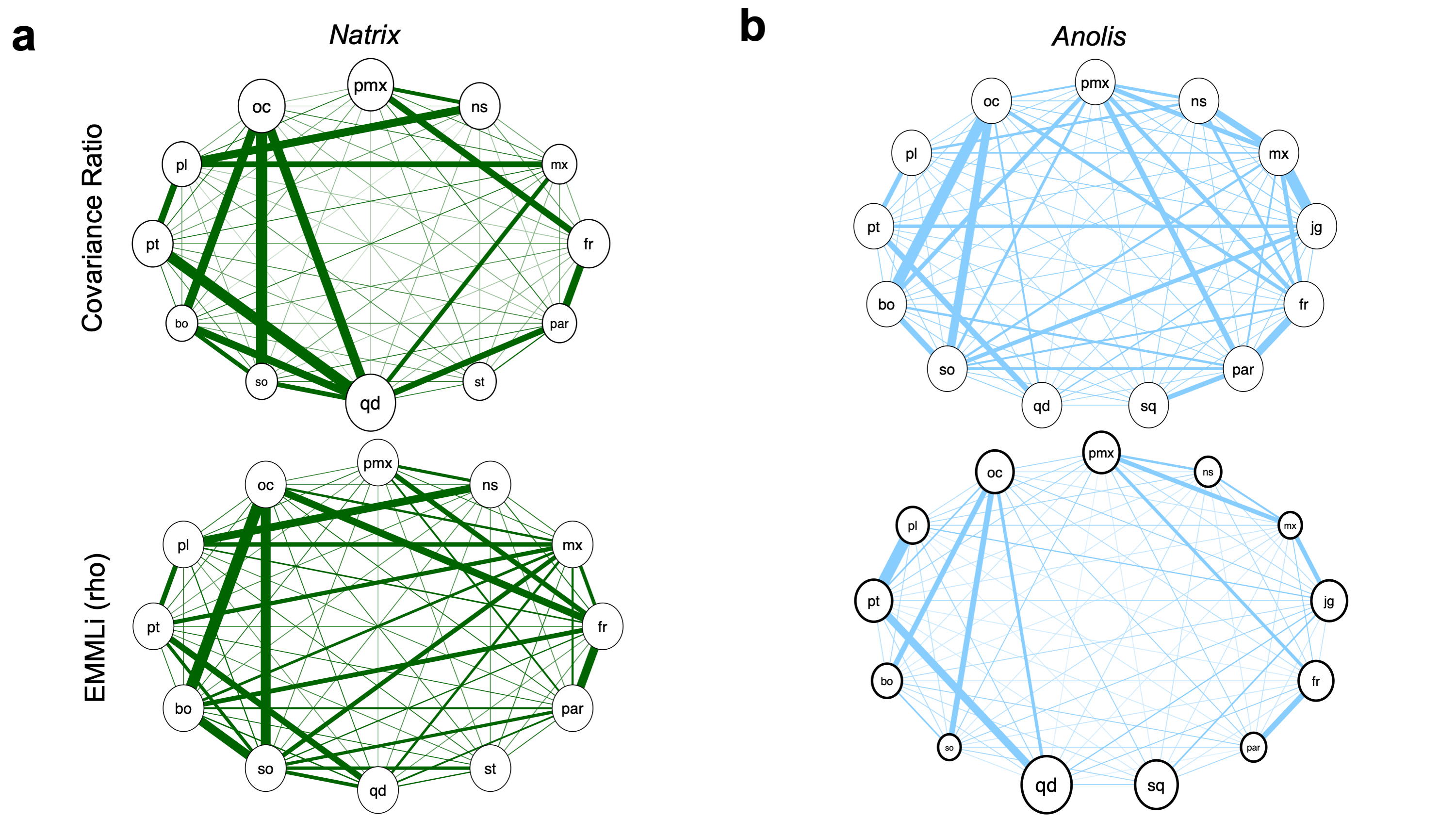

Supplement: obad022_Supplemental_Files [file obad022_supplemental_files.zip › FigS1.tiff]
